# Injury-related gaining momentum as external causes of deaths in Ethiopian health and demographic surveillance sites: evidence from verbal autopsy study

**DOI:** 10.1080/16549716.2018.1430669

**Published:** 2018-02-23

**Authors:** Kassahun Alemu Gelaye, Fasil Tessema, Befikadu Tariku, Semaw Ferede Abera, Alemseged Aregay Gebru, Nega Assefa, Desalew Zelalem, Melkamu Dedefo, Mekdes Kondal, Mesfin Kote, Mitike Molla Sisay, Wubegzier Mekonnen, Mamo Wubshet Terefe, Gashaw Andargie Biks, Firehywot Eshetu, Mulumebet Abera, Yoseph Fekadu, Gessessew Bugssa Hailu, Etsehiwot Tilahun, Yihunie Lakew

**Affiliations:** ^a^ Dabat Health and Demographic Surveillance Site, Amhara National Regional State, Gondar, Ethiopia; ^b^ Department of Epidemiology, Gilgel Gibe Health and Demographic Surveillance System, Oromia Region, Ethiopia; ^c^ Arba Minch Health and Demographic Surveillance Site, Southern Nation and Nationalities Regional State, Arba Minch, Ethiopia; ^d^ Kilte Awulalo Health and Demographic Surveillance Site, Tigray National Regional State, Mekelle, Ethiopia; ^e^ Department Reproductive Health; CDC Ethiopia, Kersa Health and Demographic Surveillance Site, Oromia Region, Ethiopia; ^f^ Butajira Health and Demographic Surveillance Site, Southern Nation and Nationalities Regional State, Addis Ababa, Ethiopia; ^g^ Center for Disease Control and Prevention, Addis Ababa, Ethiopia; ^h^ Department of Data Mangment, Ethiopian Public Health Association, Addis Ababa, Ethiopia

**Keywords:** Accidents, assault, drowning, external causes, mortality, suicide, transport, verbal autopsy, Ethiopia

## Abstract

**Background**: In Ethiopia, though all kinds of mortality due to external causes are an important component of overall mortality often not counted or documented on an individual basis.

**Objective**: The aim of this study was to describe the patterns of mortality from external causes using verbal autopsy (VA) method at the Ethiopian HDSS Network sites.

**Methods**: All deaths at Ethiopian HDSS sites were routinely registered and followed up with VA interviews. The VA forms comprised deaths up to 28 days, between four weeks and 14 years and 15 years and above. The cause of a death was ascertained based on an interview with next of families or other caregivers using a standardized questionnaire that draws information on signs, symptoms, medical history and circumstances preceding death after 45 days mourning period. Two physician assigned probable causes of death as underlying, immediate and contributing factors independently using information in VA forms based on the WHO ICD-10 and VA code system. Disagreed cases sent to third physician for independent review and diagnosis. The final cause of death considered when two of the three physicians assigned underlying cause of death; otherwise, labeled as undetermined.

**Results**: In the period from 2009 to 2013, a total of 9719 deaths were registered. Of the total deaths, 623 (6.4%) were from external causes. Of these, accidental drowning and submersion, 136 (21.8%), accidental fall, 113 (18.1%) and transport-related accidents, 112 (18.0%) were the topmost three leading external causes of deaths. About 436 (70.0%) of deaths were from the age group above 15 years old. Drowning and submersion and transport-related accidents were high in age group between 5 and 14 years old.

**Conclusion**: In this study, external causes of death are significant public health problems and require attention as one of prior health agenda.

## Background

Deaths from external causes can be unintentional (such as transport-related, falls, drowning, fires and burns, venoms, and poisons) or intentional (suicides and assaults). Injury is defined as the physical damage that results when a human body is suddenly subjected to energy in amounts that exceed the threshold of physiological tolerance [1]. The magnitude of deaths due to external causes has gained considerable public health significance. Over one million people have been killed on the world’s roads per annum globally [2]. Despite technological improvements in vehicles and road infrastructure, increasing traffic density has escalated the risks of injuries and deaths particularly to pedestrians. In Africa, the highest rate of road traffic deaths is estimated to 0.24 per 1,000 populations though there might be an under reporting due to absence of a reliable data source. Death due to road traffic accidents reached 22,786 (2.77%) of the total deaths in Ethiopia. The age-adjusted road traffic accident death rate was reported at 37.8 per 100,000 population, which is the 12^th^ highest in the world [3].

The number of child injury-related deaths was close to one million per year globally [4]. The majority of which were occurring in low- and middle-income countries. Road traffic and drowning are the leading causes of deaths among external causes of death categories. About 5 million deaths occurred worldwide each year due to injury and remains the leading cause of death and disability for all age groups. Traffic collisions, drowning, poisoning, falls or burns, and violence from assault are the commonest. The magnitude of the external causes of death significantly varies by specific cause, age, sex, and income group across countries [5,6]. Child injuries, since the age of one year, are also growing global public health significance and remains an important area of concern, and progressively contributes more to the overall rates of death until children reach adulthood [7].

A global analysis of suicide estimated a rate of 0.06 per 1,000 though its level in the WHO South-East Asia Region reached 0.16 per 1,000 [4]. In Africa, estimates of overall magnitude of suicide is uncertain [8]. Suicide deaths reached 7,228 (0.88%) of total deaths in Ethiopia. The age adjusted death rate is 14.0 per 100,000 of the population [9].

In Ethiopia, there is no reliable source of external causes of death statistics although there are sporadic attempts to capture these data by the police offices and health facilities during follow-up of medical care for injuries. In general, external causes of death in Ethiopia, the patterns and the extent of deaths based on the population level data using open dynamic cohort population are limited. Describing the patterns of mortality from external causes using VA method is supreme important for policymakers to give a primer attention as one of prior health agenda.

Hence, the Ethiopian universities research centers’ network in its six Health and Demographic Surveillance System (HDSS) sites have been capturing the occurrence of vital events including cause of deaths at a household level using an open cohort in geographically defined populations located in different parts of the country which allows reporting of external causes of deaths. The aim of this study was to describe the patterns of external causes of death among HDSS population of the six networked research centers in Ethiopia.

## Methods

### Profiles of HDSS sites

**Figure 1.**
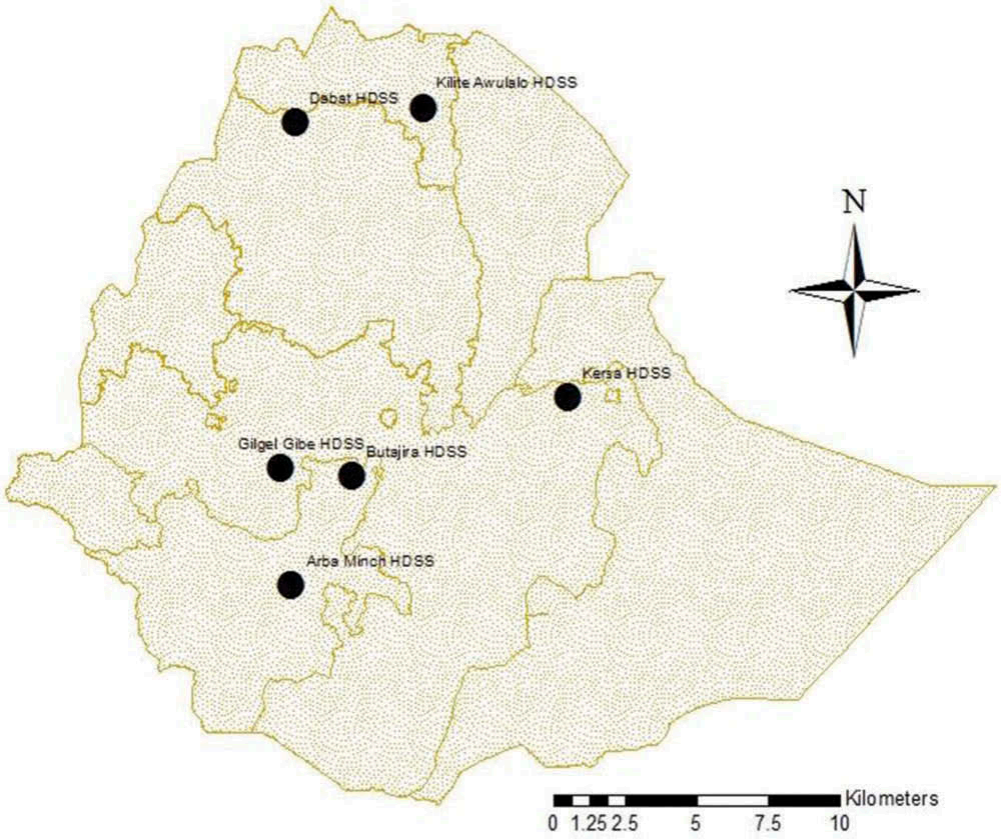
Location of health and demographic surveillance site, Ethiopia.

The Ethiopian universities research centers’ Network of HDSS sites is established to generate reliable longitudinal epidemiologic and population dynamics data in geographically defined populations located in different parts of the country. The dataset used in this study drawn from Arba Minch, Butajira, Dabat, Kilet Awulalo and Kersa HDSS sites for analyses of external causes of death (Figure 1). Arba Minch and Buta Jira HDSS are located at Southern Nations Nationality Peoples, Dabat is located at northwest of Ethiopia representing Amhara National Regional State; Gilgel Gibie HDSS is located at southwest and Kersa HDSS is located on Eastern part of Ethiopia both representing Oromia National Regional State; and Kilet Awulalo HDSS is located at northern Ethiopia representing Tigray National Regional State.

### Study design and population

The HDSS sites employed an open dynamic cohort study design. Population entered into the HDSS sites in the forms of births and in migration, and exited by deaths and out migration from the sites. The follow upstarted with a baseline census of the population in a geographically defined area and then house-to-house visits were done quarterly/biannually to capture pregnancy observations and outcomes, deaths, marriage and in-and-out migrations. Causes of death in the population from registered deaths were identified using VA procedure. Two independent physicians reviewed each completed VA form based on the WHO ICD-10 and VA code system. When the two physicians disagreed the third tie breaker physician reviewed it. If the tie breaker agreed to either one of the previous two physicians the underlying cause of death was identified, otherwise, labeled as undetermined cause of death.

### Data collection

Three sets of VA forms including, for deaths up to 28 completed days of life-neonatal period; deaths to children between 4 weeks and 14 years of age; and deaths to persons aged 15 years and above [10,11] were used. The VA data collection questionnaires were translated into local languages including *Amharic, Oromefa and Tigrenga*. The questionnaires had information on the age, sex, place of death, cause of death, short narrative history, symptoms, and health services usage in the period before death, and medical evidence available at the household about the deceased person.

The occurrence of death in the demographic surveillance area was notified by the local village based data collectors and guides. The causes of death was ascertained based on an interview with next of families or other caregivers using a standardized questionnaire that draws information on signs, symptoms, medical history and circumstances preceding death after 45 days mourning period. On the agreed day, the VA interviewer arrived at the residence of the deceased to conduct the interview with the person who was responsible for caring the deceased. In the case of absence of an appropriate interviewee up to three attempts were made to conduct an interview. VA data collectors would make sure that every section of the form would be accurately completed before the form submitted to field supervisors for scrutiny of the quality of the collected data.

The completed VA questionnaires were given to two blinded physicians and reviewed independently. When disagreements in diagnosis arose, a third physician was assigned to review the case. The final diagnosis was assigned based on agreement between the third physician and any of the two physicians. The case was considered ‘undetermined’ if all three physicians assigned a different diagnosis. Physicians label the death as ‘unspecified causes of death (VA-99)’ when it was difficult to classify based on the given information. Two physicians, trained in VA diagnosis and coding procedures assigned codes and titles for each causes of death as underlying, immediate and contributing factors independently using information in VA forms based on WHO ICD-10 and VA code system [12]. Training on the contents of questionnaires, recording, contacting close relatives and data collection procedures was given to data collectors. The training to field workers included sessions on discussion of individual symptoms, and their description in the local language for easy recognition by the respondents and demonstration of interviewing techniques by research team members.

Research team members coordinated all field activities of vital events registration and VA interview. They were also responsible for making sure that the field operations run smoothly and efficiently. Moreover, the team gave the need technical support to the entire data collectors. During the courses of the fieldwork, supervisors continually (always stand by to assist and supervise data collectors to maintain the quality of the data) visited the sites to check on the progress and sort out problems that might had been encountered by data collectors.

## Data management and analysis

The VA data and cause of deaths assigned by physician reviewers were entered using in relational database software for the three age-specific forms separately as an add-on modules of death registration. STATA version 12 software was used for cleaning and analysis the pooled data from each HDSS. The distribution of the deceased by their different background characteristics was portrayed using figures and tables. Proportionate mortality ratio was used to measure the magnitude of each cause of death from all death reported by all HDSS. It was also reported for various attributes of the deceased.

## Results

**Figure 2.**
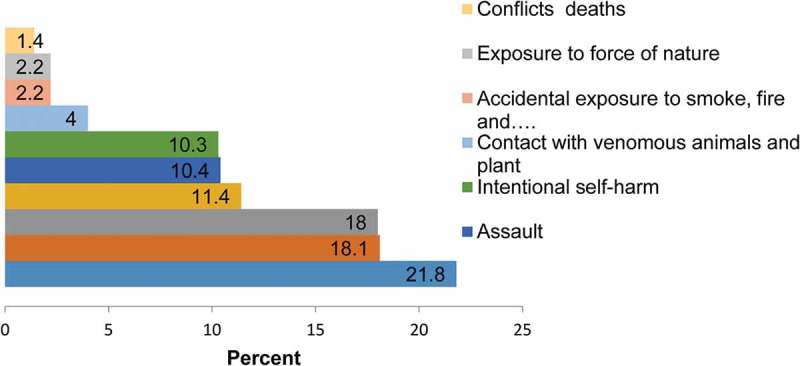
Proportion of variety of external causes of death in Ethiopian Universities Research Center HDSS sites between 2009 and 2013, Ethiopia.

**Figure 3.**
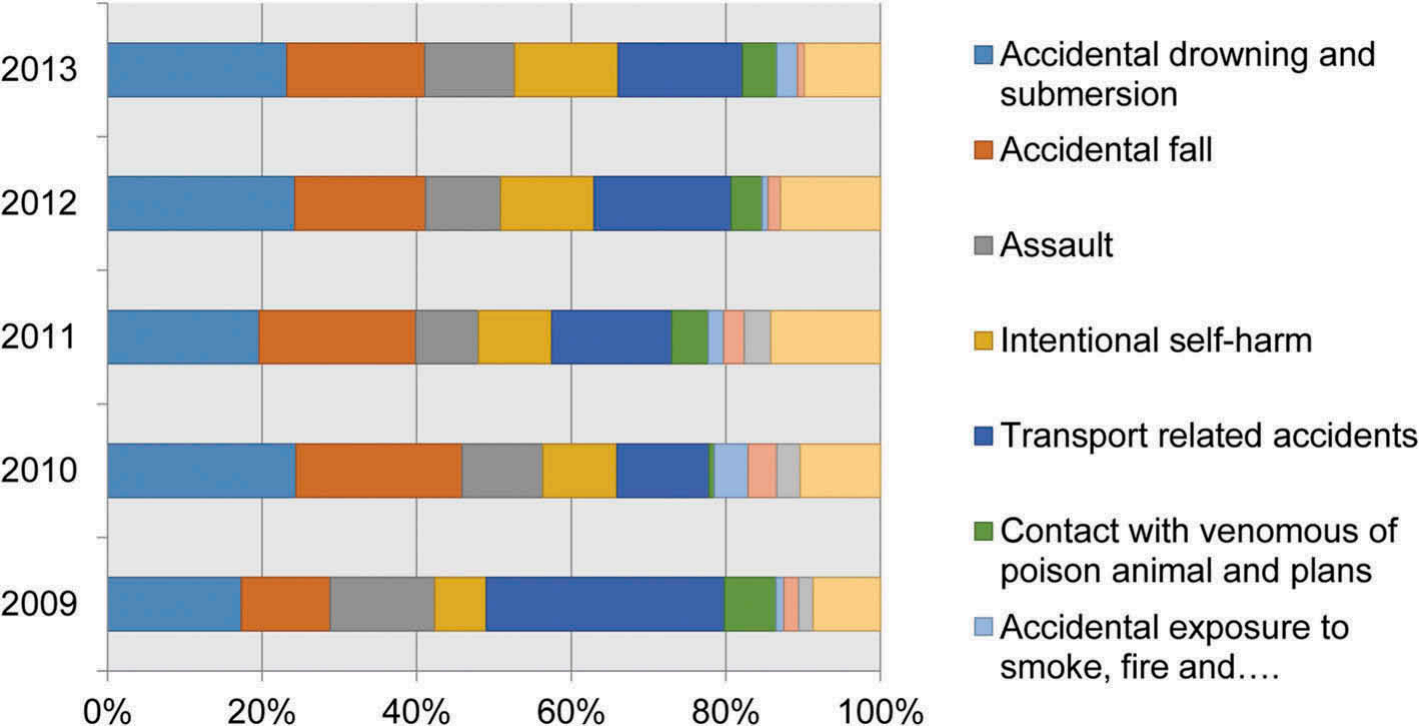
Trends of external causes of deaths at Ethiopian Universities Research Center HDSS sites between 2009 and 2013, Ethiopia.

Of the total deaths captured by the networked HDSS, 623 (6.4%) were from external causes. Among external causes of death, accidental drowning and submersion, 136 (21.8%) was the leading cause of death followed by accidental fall, 113 (18.1%) and transport-related accidents, 112 (18.0%) (Figure 2). About 85.4% (532) of mortality from external causes were from rural area. More males 418 (67.1%) were the victims of external causes of death. Majority of the deceased from external causes of death 343 (56.2%) were those who were not able to read and write. About 283 (40%) the external causes of death happened at home. Figure 3, shows the overall proportions of variety of external cause of deaths.

**Figure 4.**
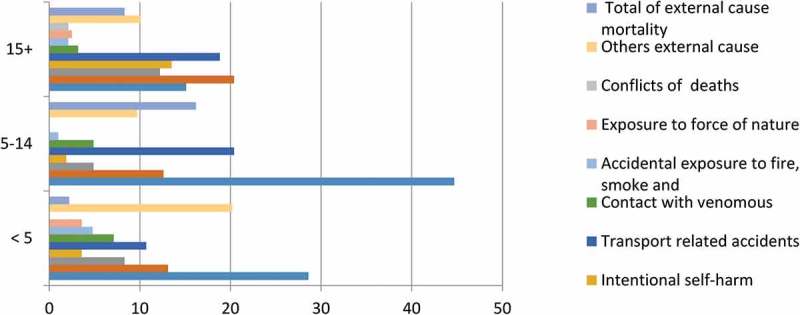
Distribution of external causes of death by age in Ethiopian Universities Research Centers HDSS sites between 2009 and 2013, Ethiopia.

Although the pattern of mortality between 2009 and 2013 from external causes was consistent, it was a bit higher in the years 2010 and 2011. Of these, deaths from accidental drowning and submersion, and accidental fall accounted 249 (40%) of the total external causes of death. Four hundred seven (56%) of the deaths from external causes occurred during 2010 through 2012 (Figure 4).

The majority of deaths due to external causes were reported from Kilete Awulalo, 150 (24.1%), Dabat, 142 (22.8%) and Gilgel Gibie, 98 (15.7%) HDSS sites. Deaths due to accidental drowning and submersion was high in Gilgel Gibie, 44 (44.9%) followed by Kilite Awulalo, 44 (29.3%). The majority of deaths from accidental fall were reported from Kilete Awulalo, 45 (30.0%) Arba Minch, 19 (21.6%) and Dabat, 24 (16.9%) HDSS sites. In Kersa HDSS site, transport-related accidents 39 (41.1%) was the major cause of death from external causes (Table 1).

**Table 1.** Distribution of deaths from a variety of external causes across Ethiopian Universities Research Centers’ HDSS sites between 2009 and 2013, Ethiopia.

	HDSS Sites (# (%))						
External causes of death	Butajira	Dabat	Gilgel Gibe	Kersa	Kilite Awlalo	ArbaMinchi	Total
Accidental drowning and submersion	11 (22)	18 (12.7)	44 (44.9)	9 (9.5)	44 (29.3)	10 (11.4)	136 (21.8)
Accidental fall	4 (8)	24 (16.9)	11 (11.2)	10 (10.5)	45 (30.0)	19 (21.6)	113 (18.1)
Assault	10 (20)	13 (9.2)	8 (8.2)	14 (14.7)	6 (4.0)	14 (15.9)	65 (10.4)
Intentional self-harm	5 (10)	22 (15.5)	9 (9.2)	5 (5.3)	11 (7.3)	12 (13.6)	64 (10.3)
Transport-related accidents	12 (24)	13 (9.2)	18 (18.4)	39 (41.1)	15 (10.0)	15 (17.0)	112 (18.0)
Contact with venomous	1 (2)	13 (9.2)	1 (1.0)	1 (1.1)	9 (6.0)	0 (0.0)	34 (5.5)
Accidental exposure to fire, smoke	0 (0)	4 (2.8)	1 (1.0)	4 (4.2)	3 (2.0)	2 (2.3)	28 (4.5)
Exposure to force of nature	1 (2)	4 (2.8)	0 (0.0)	1 (1.1)	6 (4.0)	2 (2.3)	14 (2.2)
Conflict deaths	0 (0)	8 (5.6)	0 (0.0)	0 (0.0)	1 (0.7)	0 (0.0)	9 (1.4)
Others external cause	6 (12)	23 (16.2)	6 (6.1)	12 (12.6)	10 (6.7)	14 (15.9)	71 (11.4)
Total external causes of death	50 (4.3)	142 (8.7)	98 (4.7)	95 (3.7)	150 (12.1)	88 (8.8)	623 (100)
All other causes	1,100 (95.7)	1,499 (91.3)	1,993 (95.3)	2,491 (96.3)	1,088 (87.9)	915 (91.2)	9096
Total	1150	1641	2091	2586	1238	1003	9719

Among a total (9719) of deaths in six HDSS sites, 7733 (79.7%) occurred among rural residents. When we disaggregate external causes of death by residence type, 532 (85.4%) of them happened among rural residents of the networked HDSS sites in Ethiopia. Accidental drowning and submersion, accidental fall and transport-related accidents were the top three leading external cause’s death among both rural residents and urbanites in the six networked HDSS sites of Ethiopia. However, 91 (6.3%) of external causes of death were contributed urban dwellers in the networked sites (Table 2).

**Table 2.** Distribution of deaths from external causes by urban and rural in Ethiopian Universities Research Center HDSS sites between 2009 and 2013, Ethiopia.

	Urban	Rural	Total
External causes of deaths	Number (%)	Number (%)	Number (%)
Accidental drowning and submersion	10 (11.0)	126 (23.7)	136 (21.8)
Accidental fall	12 (13.2)	101 (19.0)	113 (18.1)
Assault	11 (12.1)	54 (10.2)	65 (10.4)
Intentional self-harm	10 (11.0)	54 (10.2)	64 (10.3)
Transport-related accidents	28 (30.8)	84 (15.8)	112 (18.0)
Contact with venomous	0 (0.0)	25 (4.7)	25 (4.0)
Accidental exposure to fire, smoke	4 (4.4)	10 (1.9)	14 (2.2)
Exposure to force of nature	0 (0.0)	14 (2.6)	14 (2.2)
Conflict deaths	2 (2.2)	7 (1.3)	9 (1.4)
Others external causes	14 (15.4)	57 (10.7)	71 (11.4)
Total	91 (6.3)	532 (6.4)	623 (6.4)
All other causes	1353 (93.7)	7733 (93.6)	9,096 (93.6)
Total	1444 (100)	8265 (100)	9,719 (100)

External causes of death had its highest toll among males 418 (67.1%). The main leading external causes of death in the six networked HDSS sites were accidental drowning and submersion 135 (21.7%), accidental fall, 113 (18.2%) and transport-related accident, 112 (18.0) for both males and females (Table 3). About 436 (70.0%) of external causes of death happened among those aged 15 years and older. Accidental fall, transport-related accidents and accidental drowning and submersion remained the major external causes of death even among those aged above 15 years old. On the other hand, the main external causes of death among children aged 5–14 years old were drowning and submersion and transport-related accidents (Figure 4).

**Table 3.** Distribution of variety of external causes death by sex in Ethiopian Universities Research Center HDSS sites between 2009 and 2013, Ethiopia.

	Male	Female	Total
	Number (%)	Number (%)	Number (%)
Accidental drowning and submersion	43 (21.1)	93 (22.0)	136 (21.7)
Accidental fall	38 (18.6)	75 (17.9)	113 (18.2)
Assault	22 (10.8)	43 (10.3)	65 (10.5)
Intentional self-harm	21 (10.3)	44 (10.3)	64 (10.3)
Transport-related accidents	37 (18.1)	75 (17.9)	112 (18.0)
Contact with venomous	9 (4.4)	16 (3.8)	25 (4.0)
Accidental exposure to fire, smoke	8 (3.9)	6 (1.4)	14 (2.3)
Exposure to force of nature	2 (1.0)	12 (2.9)	14 (2.3)
Conflicts deaths	1 (0.5)	8 (1.9)	9 (1.4)
Others external cause	23 (11.3)	48 (11.5)	71 (11.4)
Total external causes	204 (4.4)	418 (8.2)	623 (6.4)
All other causes	4,397 (95.6)	4,677 (91.8)	9,096 (93.6)
Total	4,601 (100.0)	5,095 (100.0)	9,719 (100)

Dis-aggregation of total of deaths in the six HDSS sites by the place of death of the deceased revealed that 7613 of deaths happened at home. When we disentangled external causes of death by its place of occurrence, about 283 (47%) of them happened at home followed by those encountered in the field, 233 (37.4%) and in health facilities, 89 (14.3%) (Table 4).

**Table 4.** Distribution of external causes of death by place of death in the six Ethiopian Universities Research Centers Networked HDSS sites, between 2009 and 2013, Ethiopia.

	Place of Deaths			
	Home	Health Institution	Field	Total
External causes of deaths	Number (%)	Number (%)	Number (%)	Number (%)
Accidental drowning and submersion	26 (9.2)	5 (5.6)	104 (44.6)	136 (21.7)
Accidental fall	65 (23.0)	19 (21.3)	26 (11.2)	113 (18.2)
Assault	36 (12.7)	8 (9.0)	20 (8.6)	65 (10.5)
Intentional self-harm	39 (13.8)	7 (7.9)	16 (6.9)	64 (10.3)
Transport-related accident	40 (14.1)	26 (29.2)	35 (15.0)	112 (18.0)
Contact with venomous	14 (4.9)	5 (5.6)	6 (2.6)	25 (4.0)
Accidental exposure to fire, smoke	11 (3.9)	3 (3.4)	0 (0.0)	14 (2.3)
Exposure to force of nature	7 (2.5)	0 (0.0)	7 (3.0)	14 (2.3)
War deaths	2 (0.7)	1 (1.1)	6 (2.6)	9 (1.4)
Others external cause	43 (15.2)	15 (16.9)	13 (5.6)	71 (11.4)
Total external causes	283 (3.7)	89 (7.9)	233 (48.8)	623 (6.4)
All other causes	7,330 (96.3)	1,044 (92.1)	244 (51.2)	9,096 (93.6)
Total	7,613 (100.0)	1,133 (100.0)	477 (100.0)	9,719 (100)

**Table 5.** Distribution of the variety of external causes of death by educational status of the deceased in the six networked Ethiopian Universities Research Centers HDSS sites, between 2009 and 2013, Ethiopia.

	Educational status			
External causes of death	Illiterate	1–8 grade	9–12 grade	Higher
Accidental drowning and submersion	65 (19.0)	57 (27.1)	5 (10.4)	1 (11.1)
Accidental fall	82 (23.9)	25 (11.9)	5 (10.4)	0 (0.0)
Assault	34 (9.9)	27 (12.9)	3 (6.3)	0 (0.0)
Intentional self-harm	29 (8.5)	18 (8.6)	15 (31.3)	2 (22.2)
Transport-related accident	48 (14.0)	45 (21.4)	15 (31.3)	4 (44.4)
Contact with venomous	18 (5.2)	6 (2.9)	1 (2.1)	0 (0.0)
Accidental exposure to fire, smoke	11 (3.2)	3 (1.4)	0 (0.0)	0 (0.0)
Exposure to force of nature	5 (1.5)	7 (3.3)	0 (0.0)	1 (11.1)
War deaths	4 (1.2)	5 (2.4)	0 (0.0)	0 (0.0)
Others external cause	47 (13.7)	17 (8.1)	4 (8.3)	1 (1.11)
Total external causes	343 (4.4)	210 (19.7)	48 (21.2)	9 (12.5)
All other causes	7,538 (95.6)	858 (80.3)	178 (78.8)	63 (87.5)
Total	7,881 (100)	1,068 (100)	226 (100.0)	72 (100)

The death toll from accidental drowning and submersion and fall decreased as educational level increased in the six networked HDSS sites in Ethiopia. Assault was higher among those who enrolled in the primary level of education. On the other hand, intentional self-harm increased as level of education increased in Ethiopian networked HDSS sites. Deaths from transport-related accident were high among high school and above educated individuals in the study area. Contact with venomous was rather higher among those who were not able to read and write (Table 5).

## Discussion

In this study, the majority of external causes of deaths affected rural residents and males. Accidental drowning and submersion, accidental fall and transport-related accidents were the top leading external causes of death. Residents of Kilete Awulalo and Dabat HDSS sites were reported that suffered more from external causes of death compared with other HDSS sites. Higher number of accidental drowning and submersion and accidental fall were reported in Kilete Awulalo and Dabat HDSS sites since the study areas have rigged land surfaces that expose to fall from terrains. On the other hand, highest number of drowning and submersion was reported from Gilgel Gibe HDSS which established in villages around a hydro-electric dam which might expose children for such an injury. Kersa HDSS site, however, established in high transport corridor in Eastern Ethiopia which documented relatively highest number of deaths due to transport-related accidents. External causes of deaths were higher among those aged above 15 years old which might be attributed to the nature of adults to engage in farming and other physically demanding jobs performed in sliced plots of land that may cause conflict among neighbors. On the other hand, drowning and submersion and transport-related accidents had its heaviest toll among children aged 5 and 14 years old that would be left alone in the field with animals when adult family members were busy in their farming routine activities.

This study documented that about 6.4% of deaths in the six networked HDSS centers were attributed to external causes of death in Ethiopia. A similar finding was reported by the Mozambican study which claimed the lives of approximately 11% adults and another study in Abhoynagar which reported an injury and other external causes of death to account for another 7.5–7.7% of total deaths [13] while in rural Bangladesh, injury and other external causes of death accounted for another 5% of the deaths [12].

On the other hand, this study revealed that more males (67.1%) were reported to die off external causes compared with females which is similar to findings of a study done in South Africa [14]. Mortality due to injury was also reported to vary by age and gender in Tanzania [13].

This study revealed that about 40% of external causes of death happened at home. The findings suggest that the health managers and policy-makers of Ethiopia should give more attention to make awareness on more occurrence of external causes of death in the homes of Ethiopians and the health sector should give priority in designing prevention and management of external causes of deaths for rural people of the country.

In this study, accidental drowning and submersion, accidental fall and transport-related accidents were the top three leading external causes of deaths. A former study in the Kilete Awulalo, HDSS site reported a similar finding of accidental drowning and submersion to be the leading external causes of death [15]. Rural residents in Ethiopia should be aware on the dangers of water bodies and rough terrains around them. The health sector and its partners should strive to educate community members on the risks of their environment in inflicting external causes of death. This study also revealed that deaths to road traffic accident claimed the lives of many residents in the six networked HDSS sites which has been repeatedly reported by electronic and print media in the country. Children, between 5 to 15 years old died by accidental drowning and submersion. In a rural population in KwaZulu-Natal, South Africa, injury accounted 8% of all specific causes of death. In Bangladesh, causes of death were dominated by childhood drowning and by transport-related deaths and intentional injuries elsewhere [10].

The majority of the deaths were from the age group above 15 years old. These age groups are mostly productive age groups where all the livelihoods are depend on and have a frequent exposure of the external causes of deaths comparing with the counterparts. Deaths due to external causes are an important component of overall deaths which contributed for many premature deaths [8]. This information helps to set targeted external causes prevention. The surveillance system on external causes would be very important to point out the magnitude of deaths from external causes. National injury data administrators should be established for the provision of comprehensive injury reports and for serving the needs of key stakeholders in injury prevention [11]. Most injuries are preventable, policy makers need to institute measures to address the issue[16].

Of the total deaths in six HDSS sites, the majority of deaths due to external causes were reported from Kilete Awulalo, 150 (24.1%), Dabat, 142 (22.8%) and Gilgel Gibie, 98 (15.7%) HDSS sites. Deaths due to accidental drowning was high in Gilgel Gibie, 44 (44.9%) followed by Kilite Awulalo, 44 (29.3%). This would be possibly that Gilgel Gibie HDSS site is located nearby Gilgel Gibie Hydro Electric Dam. However, in the Kilite Awulalo HDSS site, there are rivers which could be the reason for the toll of accidental drowning and submersion. The majority of deaths from accidental fall were reported from Kilete Awulalo, 45 (30.0%) Arba Minch, 19 (21.6%) and Dabat, 24 (16.9%) HDSS sites. The population mainly live and work in hilly, deep valley and mountainous areas. In Kersa HDSS site, transport-related accidents 39 (41.1%) was the major cause of mortality from external causes. In this HDSS site, manly traffic accidents occurred due to night time driving and the drivers have been exposed of substances such as Khat. External causes of deaths were high above 15 years old. These age groups were mainly prone as many of them were reproductive age group whom the responsibility of the households shouldered on them that exposed them to transport-related accidents, accidental fall and others as well. Geographic location, age, and sex are major determinants not only of overall external cause of deaths but also of specific cause categories. In some cases, geographic location appeared to play a direct role. A number of the mortality burdens revealed clearly constitute grounds for public health actions [10].

External causes of death represent a relatively easy option for assigning cause of death via VA. This may be true for a proportion of deaths from external causes, for example, sudden fatalities with no complicating factors. That all deaths from external causes could be a witnesses who can be traced for VA interviews. In this study, there might also have been some difficulties in extracting all the necessary data items correctly, particularly for details of injuries contained in narratives [16]. Calculating Kappa statistics was important to determine the agreement between two Physicians in assigning the causes of deaths where did not practiced in this study. Verbal autopsy based methods enabled the timely measurement of changing trends in cause-specific mortality to provide policymakers with the much-needed information to allocate resources to appropriate health interventions.

## Conclusion

In this study, accidental drowning and submersion, accidental fall and transport-related accidents were the top three leading external causes for deaths. External causes of death varied across sites. Deaths due to accidental drowning and submersion was high in Gilgel Gibie, and Kilite Awulalo HDSS sites. The majority of deaths from accidental fall were reported from Kilete Awulalo, Arba Minch, and Dabat HDSS sites. In Kersa HDSS site, transport-related accidents was the major cause of deaths from external causes. External causes of deaths are one of the major public health problems in six HDSS sites. Evidence-based interventions and community awareness are needed for lowering such deaths.

## Paper context

In Ethiopia, there is no reliable source of external causes of death statistics. Describing patterns of mortality from external causes using VA method is important. Cause of a death was ascertained based on an interview with next of families or caregivers using a standardized questionnaire. Drowning and submersion, fall, and transport-related accidents were the leading external causes of deaths. External causes of death are significant public health problem and requires attention as one of prior health agenda; U.S. President’s Emergency Plan for AIDS Relief [GH001039-01].

## References

[1] Baker SP, O’neill B, M j G, et al The injury fact book. 2nd ed. Lexington (MA): Lexington Books; 1992.

[2] World Health Organization The global status report on road safety. Geneva: World Health Organization; 2013.

[3] World Health Organization World report on child injury prevention. Geneva: World Health Organization; 2008. 26269872

[4] World Health Organization Global health estimates technical paper. Geneva: WorldHealth Organization; 2008.

[5] Global Health Observatory (GHO) data Mortality and global health estimates. 2012.

[6] Mars B BS, Hjelmeland H, Gunnell D. Suicidal behaviour across the African continent: a review of the literature. BMC Public Health. 2014;14:606. 24927746 10.1186/1471-2458-14-606 PMC4067111

[7] World Health Organization Verbal autopsy standards: The2012 WHO verbal autopsy instrument. 2012.

[8] Alam N, Chowdhury HR, Das SC, et al Causes of death in two rural demographic surveillance sites in Bangladesh, 2004–2010: automated coding of verbal autopsies using InterVA-4. Glob Health Action. 2014;29 (7):25511. 10.3402/gha.v7.25511 PMC4220132 25377334

[9] Sartorius B, Kahn K, Collinson MA, et al Dying in their prime: determinants and space-time risk of adult mortality in rural South Africa. Geospat Health. 2013;7:237. 23733287 10.4081/gh.2013.83 PMC3725424

[10] Sankoh O, Byass P Cause-specific mortality at INDEPTH Health and Demographic Surveillance System sites in Africa and Asia: concluding synthesis. Glob Health Action. 2014;29 (7):25590. 10.3402/gha.v7.25590 PMC4220138 25377341

[11] Kissera R, Latarjetb J, Bauerc R, et al Selected papers from the European Conference on Injury Prevention and Safety Promotion. Int J Inj Contr Saf Promot. 2009;16:2. 10.1080/17457300902836689 19941205

[12] Alam N, Chowdhury HR, Bhuiyan MA, et al Causes of death of adults and elderly and healthcare-seeking before death in Rural Bangladesh. J Health, Popul Nutr. 2010;28:520–9. 20941904 10.3329/jhpn.v28i5.6161 PMC2963775

[13] Ayuurebobi K, Masanja H, Kellerman R, et al Risk factors for injury mortality in rural Tanzania: a secondary data analysis. BMJ Open. 2012;2:e001721. 10.1136/bmjopen-2012-001721 PMC3533022 23166132

[14] Herbst AJ, Author C, Mafojane T, et al Verbal autopsy-based cause-specific mortality trends in rural KwaZulu-Natal, South Africa, 2000-2009. Popul Health Metrics. 2011;9:47. 10.1186/1478-7954-9-47 PMC3160940 21819602

[15] Weldearegawi B, Ashebir Y, Gebeye E, et al Emerging chronic non-communicable diseases in rural communities of Northern Ethiopia: evidence using population-based verbal autopsy method in Kilite Awlaelo surveillance site. Health Policy Plan. 2013;28:891–898. 23293101 10.1093/heapol/czs135

[16] Streatfieldsw PK, Khan WA, Bhuiya A, et al Mortality from external causes in Africa and Asia: evidence from INDEPTH Health and Demographic Surveillance System Sites. Glob Health Action. 2014;29 (7):25366. 10.3402/gha.v7.25366 PMC4220124 25377327

